# Contribution of Cell Elongation to the Biofilm Formation of *Pseudomonas aeruginosa* during Anaerobic Respiration

**DOI:** 10.1371/journal.pone.0016105

**Published:** 2011-01-18

**Authors:** Mi Young Yoon, Kang-Mu Lee, Yongjin Park, Sang Sun Yoon

**Affiliations:** 1 Department of Microbiology, Yonsei University College of Medicine, Seoul, Republic of Korea; 2 Brain Korea 21 Project for Medical Sciences, Yonsei University College of Medicine, Seoul, Republic of Korea; 3 Research Institute of Bacterial Resistance, Yonsei University College of Medicine, Seoul, Republic of Korea; Tulane University, United States of America

## Abstract

*Pseudomonas aeruginosa*, a gram-negative bacterium of clinical importance, forms more robust biofilm during anaerobic respiration, a mode of growth presumed to occur in abnormally thickened mucus layer lining the cystic fibrosis (CF) patient airway. However, molecular basis behind this anaerobiosis-triggered robust biofilm formation is not clearly defined yet. Here, we identified a morphological change naturally accompanied by anaerobic respiration in *P. aeruginosa* and investigated its effect on the biofilm formation *in vitro*. A standard laboratory strain, PAO1 was highly elongated during anaerobic respiration compared with bacteria grown aerobically. Microscopic analysis demonstrated that cell elongation likely occurred as a consequence of defective cell division. Cell elongation was dependent on the presence of nitrite reductase (NIR) that reduces nitrite (NO_2_^−^) to nitric oxide (NO) and was repressed in PAO1 in the presence of carboxy-PTIO, a NO antagonist, demonstrating that cell elongation involves a process to respond to NO, a spontaneous byproduct of the anaerobic respiration. Importantly, the non-elongated NIR-deficient mutant failed to form biofilm, while a mutant of nitrate reductase (NAR) and wild type PAO1, both of which were highly elongated, formed robust biofilm. Taken together, our data reveal a role of previously undescribed cell biological event in *P. aeruginosa* biofilm formation and suggest NIR as a key player involved in such process.

## Introduction

*Pseudomonas aeruginosa* propagates as complex, highly organized communities known as biofilms in the environment and during various infections [1]. Bacterial biofilms are the most common causes of chronic *P. aeruginosa* infection and are difficult to eradicate. For example, *P. aeruginosa* in biofilms have been reported to be more resistant to H_2_O_2_ [2], a range of antibiotics [3] and various heavy metals [4]. Moreover, bacteria grown as biofilms are more resistant to neutrophil-mediated host defenses than are their free-living planktonic counterparts [5].

*P. aeruginosa* can generate sufficient energy even under anaerobic conditions through respiration using nitrate (NO_3_^−^) or nitrite (NO_2_^−^) as terminal electron acceptors [6], [7]. It was demonstrated that (i) the oxygen potential of abnormally altered CF airways, which are highly susceptible to chronic *P. aeruginosa* infection, is extremely low [8] and (ii) that nitrate (NO_3_^−^) and nitrite (NO_2_^−^) are present in large quantities inside patient airways [9], [10]. Given the fact that PAO1, when grown under anaerobic conditions, becomes more resistant to antibiotic treatment [11], these findings provided a novel insight that reflects *P. aeruginosa* infection dynamics in CF airways and suggested to change the way of confronting *P. aeruginosa* airway infection.

Genome-wide microarray analysis revealed that the expression of a total of 691 genes (12% of the genome) was modulated upon anaerobic growth demonstrating that *P. aeruginosa*, as a versatile organism, can actively adapt itself to growth with alternative electron acceptors [12]. Cellular events that specifically occur during anaerobic respiration include decreased production of pyocyanin [13] and elastase [14], increased levels of alginate secretion [8], consistent production of sublethal levels of NO [6], [15], [16] and enhanced biofilm formation [17], [18]. Although the molecular basis of these anaerobiosis-induced changes is not fully understood, it is likely that *P. aeruginosa* may increase its survival fitness inside the patient airway, especially at the chronic stage, by reducing the production of virulence factors and increasing the capability to form biofilm. Consistent with this notion, *P. aeruginosa* isolates from chronically infected patients exhibited weaker elastase activity than those recovered from colonized CF patients or from pediatric patients without CF [19] and possessed mutations in *lasR* gene [20].

Biofilm formation is a developmental process by which bacteria undergoes significant phenotypic and genetic changes including the acquisition of antibiotic resistance and modulation of growth properties [21], [22], [23]. It is of particular interest that *P. aeruginosa* forms more robust biofilm under anaerobic respiration [17], because this demonstrates a resistant mode of bacterial proliferation under a condition that specifically represents the abnormal CF airway. Among many determinants that contribute to biofilm maturation, initial surface attachment [17], [24] and secretion of matrix molecules [25] are often considered to be critical. In this study, a unique morphological change that occurs specifically under anaerobic growth and thus influences biofilm formation was observed for the first time in *P. aeruginosa*. Wild type PAO1 was highly elongated during anaerobic respiration and an elongation-defective mutant formed very weak biofilm. Cell elongation was attributable to the organism's response to nitric oxide (NO). Ultimately, this observation may form a basis for deciphering the mechanisms by which *P. aeruginosa* modulates its pathogenic properties under anaerobic growth condition.

## Materials and Methods

### Bacterial strains and growth conditions

Wild type *P. aeruginosa* strain PAO1 and mucoid strain FRD1 have been previously described [26]. *P. aeruginosa* transposon insertion mutants, Δ*narG*, Δ*nirS*, Δ*norC* and Δ*anr* mutant strains were purchased from a *P. aeruginosa* transposon mutant library (http://www.genome.washington.edu/UWGC/pseudomonas) and sequence verified. L-broth (LB, 10 g tryptone, 10 g NaCl, 5 g yeast extract per liter) was used to grow bacteria. Anaerobic bacterial growth was achieved using a GasPak anaerobic system (Becton, Dickinson and Company, Franklin Lakes, NJ) or in a Coy anaerobic chamber (Coylab Inc. Grass Lake, MI). The gas composition inside the anaerobic chamber was a trimix of nitrogen, hydrogen, and carbon dioxide (90, 5, and 5%, respectively). Dry anaerobic indicator strip (Becton, Dickinson and Company) was used to confirm the generation of anaerobic atmosphere inside the jar. In addition, no detectable growth of PAO1 in plain LB was confirmed in each anaerobic experiment. To support anaerobic growth, KNO_3_ or NaNO_2_ (Sigma-Aldrich) was added to the medium. When required, pH of the LB medium was adjusted to pH 7.8 using 50 mM phosphate.

### Scanning Electron Microscope and cell length measurement

Bacterial cells were visualized by scanning electron microscope (SEM) and confocal microscope. For the sample preparation of SEM, bacterial suspension was fixed with PBS containing 2% glutaraldehyde and 0.1% paraformaldehyde for 2 hrs and stained with 1% OsO_4_. Samples were then coated with gold by an ion sputter (IB-3 Eiko, Japan) and examined with a scanning electron microscope (FE SEM S-800, Hitachi, Japan) at an acceleration voltage of 20 kV. Images were processed with ESCAN 4000 software (Bummi Universe Co., LTD, Seoul, Korea). For the cell length measurement, more than 100 straight-lined cells were randomly chosen in the digitized SEM images and distance between two ends was automatically calculated.

### Confocal microscopy

Differential Interference Contrast (DIC) images were acquired using a confocal laser scanning microscope (FV-1000; Olympus Optical Co. Ltd., Japan) and its operating software, FV10-ASW (ver. 02.01). Prior to the image analysis, aliquots of bacterial cultures were washed with PBS and mounted in wells of 8-well Lab-Tek™ chambered coverglass (cat. no. 155411, Nalge Nunc International, Rochester, NY). After scanning with 488 nm laser at a sampling speed of 12.5 µs/pixel, a 640×640 pixel, 12-bit image (57.51 µm×57.51 µm) was acquired. UPLSAPO 100XO (Olympus) objective lens was used for the bacterial cell image analysis. Images were saved as a TIF file with embedded 5 µm scale bar.

For the membrane visualization, PAO1 grown in LB plus 0.4% NO_3_^−^ to the early stationary phase under aerobic or anaerobic condition was washed with PBS and the cells were resuspended in 200 µl of PBS containing 10 µM of the lipophilic membrane dye TMA-DPH (Invitrogen Corp. Carlsbad, CA). Again, 8-well Lab-Tek™ chambered coverglass (cat. no. 155411, Nalge Nunc International) was used to mount samples on the objective lens. Image acquisition was conducted as described for the DIC image analysis except that samples were scanned at 405 nm and emissions were collected at 461 nm.

For the nucleoid staining, Syto 9 green fluorescent dye (Invitrogen Corp. Carlsbad, CA) was used at 10 µM final concentration. To capture the green fluorescence, samples were scanned at 488 nm and emission was detected through a 520 nm band filter. The DIC and green fluorescence images were collected simultaneously.

### Counting the cluster formation in the planktonic culture of *P. aeruginosa* strains

To count the number of clusters, 20 µl of each bacterial culture was pipetted onto the slide glass. A 22×40 mm coverslip (Paul Marienfeld GmbH & Co. KG, Lauda-Koenigshofen, Germany) was then placed over the rectangular area of the slide glass. Nikon SE optical microscope (Nikon Vision Co., Ltd) was used to view and count the number of clusters in the sample. Cluster counting was performed while the objective lens was manually moved from the top left to the bottom right region of the coverslip. Aggregates that consist of more than 10 cells were considered as clusters.

### Biofilm assays

The ability of *P. aeruginosa* to develop biofilm was assessed with a modified microtiter plate assay as described previously [24]. Briefly, *P. aeruginosa* aerobic preculture grown in LB was inoculated (1:100 dilution) into the anaerobic culture media (i.e. LB containing NO_3_^−^ or NO_2_^−^) in 24-well or 96-well plates and incubated anaerobically for 18 hrs at 37°C without agitation. Biofilm formed by *P. aeruginosa* strains was stained with 0.1% crystal violet (CV) and the stained CV was dissolved in 95% ethanol for measurement of the absorbance at 540 nm. Because the capacity to form biofilm is proportional to the bacterial cell growth, OD_540 nm_ was normalized with cell mass determined by measuring OD_600 nm_. To test the effect of antibiotic treatment on the biofilm formation, sub-MIC concentrations of carbenicillin, tobramycin or ciprofloxacin were added to the aerobic PAO1 culture in 96-well plates. The minimal inhibitory concentration (MIC) of selected antibiotics was determined as described previously [27].

### Quantitative real time-PCR (qRT-PCR) analysis

Aliquots (1.5 ml) of bacterial cell cultures were harvested by spinning down at 14,000 rpm for 5 min. Pelleted cells were resuspended with 1 ml of Trizol (Invitrigen) and then 0.2 ml of chloroform was added. After incubating 5 min at room temperature, Trizol-chloroform mixture was centrifuged for 10 min at 4°C to separate the aqueous phase containing RNA. The rest of RNA purification steps were carried out using RNeasy kit (Qiagen) following the manufacturer's instruction. The extracted RNA samples were subjected to PCR to verify the absence of contaminating DNA. The resulting RNA samples were quantified using a Nanodrop spectrophotometer (model no. ASP2680, CellTAGen Inc., Seoul Korea). For cDNA synthesis, 2 µg RNA template was mixed with 1 µl of 100 pmoles/ µl random primer (5′-NSNSNSNSNS-3′, where N = A,T,C, or G and S = C or G) and dNTP mix in 15 µl total volume. The mixture was then treated at 65°C for 5 min, followed by 5 min incubation on ice. Next, one unit of Primescript reverse transcriptase (Takara Bio Inc., Shiga, Japan) was added with 1 µl of 5x primescript reaction buffer and 13 µl DEPC-treated water. The mixture was then incubated at 42°C for 1 hr and at 70°C for 15 min. Real time PCR reaction was monitored using StepOne Real-time PCR system (Applied Biosystems, Carlsbad, CA). For the reaction, SYBR premix Ex Taq (Takara) was used following the manufacturer's instructions. Primer sets used to amplify cDNA are listed in Table S1. The PCR cycle was 95°C for 10 min and 40 cycles of 95°C for 20 s and 60°C for 20 s, followed by 95°C for 15 s and 60°C for 1 min. Transcript levels of *rpoD* gene were similar in cells grown by either aerobic or anaerobic respiration and thus, used for the normalization.

#### Statistical analysis

Data are expressed as mean ± SEM (standard error of mean). An unpaired Student's *t*-test was used to analyze the data. A *p*-value of <0.05 was considered statistically significant. All the experiments were repeated for reproducibility.

## Results

### Cell elongation occurs in PAO1 grown by anaerobic respiration using NO_3_as an alternative electron acceptor

**Figure 1:**
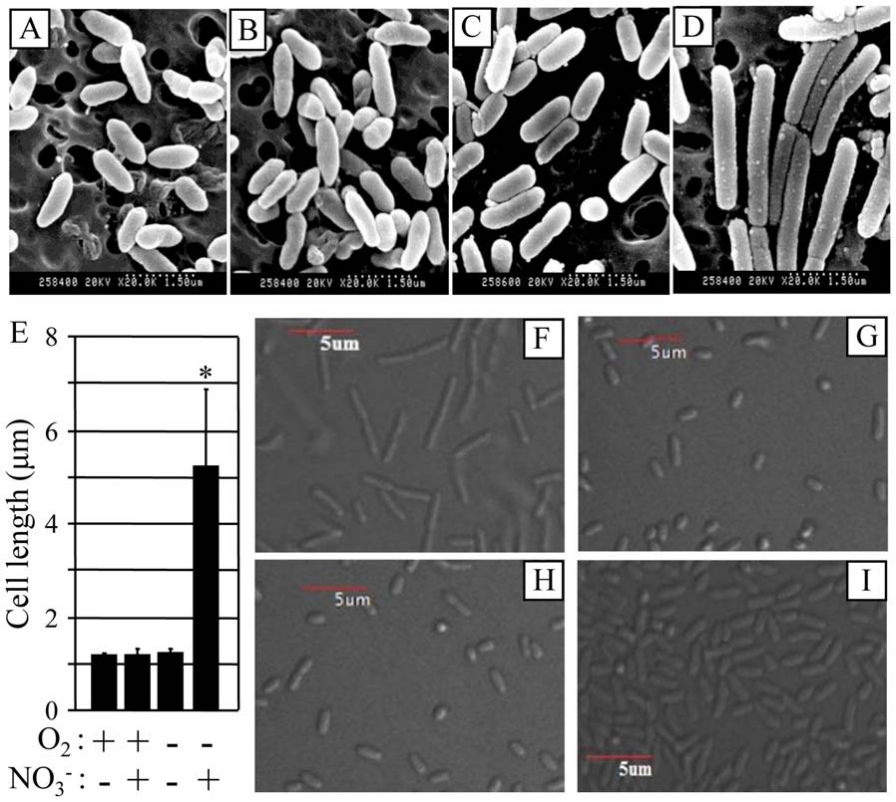
NO_3_^−^ respiration-induced cell elongation of PAO1. (**A–D**) Scanning electron microscope (SEM) images of PAO1 grown in LB (**A** and **C**) or LB containing 0.4% NO_3_^−^ (**B** and **D**) either aerobically (**A** and **B**) or anaerobically (**C** and **D**). Cells were grown for 15 hours prior to processing for SEM. The images were acquired at a magnification of 20,000 and scale bar of 1.5 µm is indicated at the bottom right. (**E**) Cell length was determined with software-aided distance measurement as described in experimental procedures. For statistical significance, more than 100 cells were selected for measurement in each image and mean ± SEM (standard error of mean) was presented. *p<0.001 vs. cells shown in the other three images. (**F–H**) DIC images of PAO1 (**F**), Δ*norC* (**G**) and Δ*anr* (**H**) grown in LB+0.4% NO_3_^−^ under anaerobic environment. (**I**) DIC image of PAO1 grown in LB +30 mM arginine under anaerobic environment. Acquisition of DIC images was conducted as described in materials and methods.

Understanding cell biological features of *P. aeruginosa* that occur specifically upon anaerobic respiration would provide a better insight into the bacterial pathogenic mechanisms under such condition. To address this important question, we first investigated cellular morphology of PAO1 grown aerobically or anaerobically by scanning electron microscope. When grown in LB with aeration, PAO1 exhibited normal rod shape morphology with a cell length of ∼1.2 µm (Fig. 1A). The addition of 0.4% NO_3_^−^ to the culture medium did not cause any detectable change in cell shape (Fig. 1B). In contrast, PAO1 grown by NO_3_^−^ respiration under anaerobic condition was highly elongated (Fig. 1D) compared to aerobically grown cells. Software-aided cell length measurement clearly demonstrated that cells are highly elongated upon anaerobic NO_3_^−^ respiration (∼5.2 µm vs. ∼1.2 µm, Fig. 1E). Cell elongation did not occur in PAO1 incubated anaerobically for the same period of time in growth medium that lacked NO_3_^−^ (Fig. 1C). No discernable growth was observed in this particular culture further proving that the presence of alternative electron acceptor is crucial for anaerobic growth in *P. aeruginosa* (Fig. S1, hatched bar). In addition, no similar elongation was observed in anaerobic cultures of mutant bacteria disrupted in nitric oxide (NO) reductase (Δ*norCB*, Fig. 1G) or ANR, one of the master anaerobic transcriptional regulators (Δ*anr*, Fig. 1H). These two mutant strains were defective in anaerobic respiration and thus no anaerobic growth was detected in previous works [6], [28]. It was also reported that *P. aeruginosa* can support, to a lesser extent, anaerobic growth by arginine fermentation [29]. As shown in Fig. 1I, no elongation was observed in cells grown by arginine fermentation. Together, these results suggest that cell elongation is caused by an active bacterial response to anaerobic respiration and not a consequence of bacterial exposure to anaerobic environments and arginine fermentation.

### Cell elongation is likely caused by defective cell division

**Figure 2:**
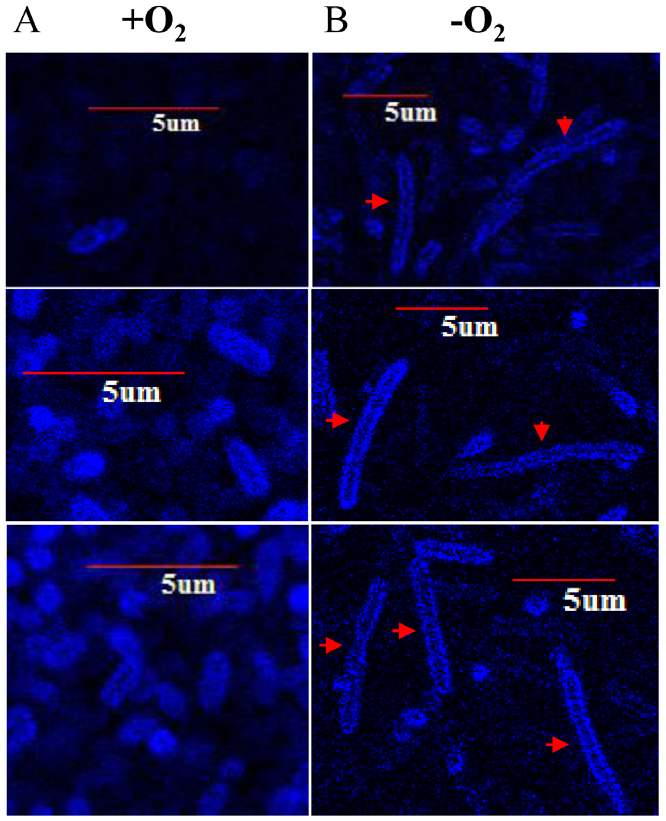
Confocal microscopic image analysis of cell membrane of elongated *P. aeruginosa*. PAO1 grown in LB+0.4% NO_3_^−^ either aerobically (**A**) or anaerobically (**B**) was stained with 10 µM TMA-DPH for the visualization of cell membrane. Three different images per sample are displayed. 5 µm scale bars were incorporated to clearly compare the cell length between two cultures. Red arrows indicate highly elongated cells. Image analysis was performed as described in materials and methods.

Fig. 1D revealed that most of elongated cells are free of invaginated cell wall pattern, which may indicate that the formation of septal peptidoglycan [30] is suppressed in PAO1 grown by anaerobic respiration. To gain a better idea of the membrane structure of elongated cells, we visualized bacterial cells stained with TMA-DPH, a lipophilic dye that specifically binds to cell membrane and thus outlines the cell periphery [31]. As shown in Fig. 2B, PAO1 grown by anaerobic respiration was highly elongated compared with that grown by aerobic respiration (Fig. 2A) further validating the results described in Fig. 1. It is of interest that continuous staining throughout the entire cell envelope was observed in elongated bacteria as indicated with red arrows (Fig. 2B). Moreover, no clear pattern of cleavage furrow formation was detected in the same cell images. This result strongly suggests that normal cellular machinery for the cell division is affected in these anaerobically grown bacteria.

**Figure 3:**
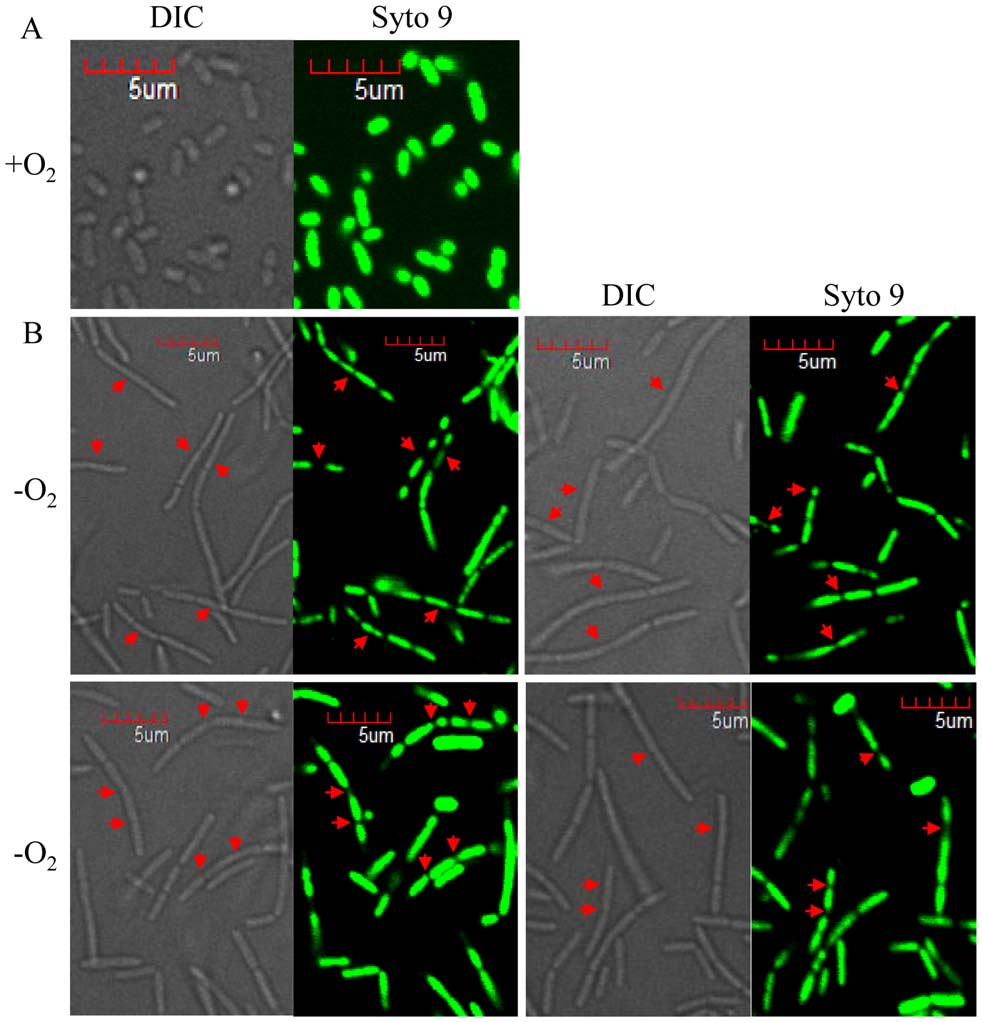
Elongated cells contain multiple nucleoids. PAO1 grown in LB+0.4% NO_3_^−^ either aerobically (**A**) or anaerobically (**B**) was stained with 10 µM Syto9, a green fluorescent dye that specifically binds to nucleic acid. Stained cells were analyzed by confocal microscopy as described in materials and methods. Simultaneously collected DIC images were shown together side by side to display the identical field of cells. Red arrows indicate the same location in a pair of cells in DIC and Syto 9 green fluorescent images.

It was reported that inhibition of chromosomal DNA replication by UV irradiation caused a cell elongation phenotype in *E. coli* [32]. Therefore, we sought to examine whether or not the replication of chromosomal DNA occurred normally in such elongated cells. To address this issue, we stained the cells with syto 9, a cell-permeant green fluorescence dye that specifically stains nucleic acid. In aerobically grown and thus rod-shaped cells, fluorescent signal was detected in the entire area of cell further suggesting that bacterial genome is not limited within a membrane-enclosed suborganelle (Fig. 3A, O_2_). Interestingly, multiple segregating nucleoids were observed in most of elongated cells (Fig. 3B, −O_2_). When we compared identical fields of images acquired by DIC or syto 9 staining side by side, distinctly segregated nucleoids were detected in elongated cells (red arrows in Fig. 3B). Together, these image analyses strongly suggest that cell elongation is due not to defects in DNA replication or chromosome segregation, but to the incomplete septum formation.

**Figure 4:**
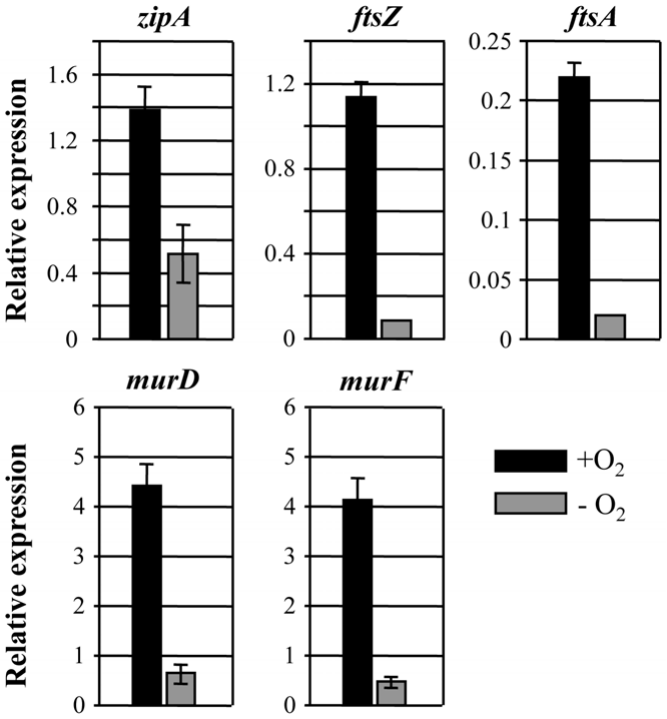
Quantitative RT-PCR analysis of genes involved in cell division and peptidoglycan synthesis. qRT-PCR was conducted on cDNA synthesized from 2 µg total RNA extracted from PAO1 grown either aerobically (black bars) or anaerobically (gray bars). Transcript levels of 5 genes indicated on top of each graph were normalized with levels of the *rpoD* transcript. Three independent experiments were performed and values of mean ± SEM are displayed in each bar.

Next, we compared the transcript levels of representative genes involved in cell division and cell wall synthesis by quantitative real time PCR (qRT-PCR). We first analyzed *zipA* (PA1528), *ftsZ* (PA4407) and *ftsA* (PA4408), because formation of Z-ring, which requires the cooperative assembly of FtsZ, FtsA and ZipA at the mid-cell region is essential for the initiation of cell division process [33]. Transcript level of *rpoD* gene, which exhibited almost identical level of expression under aerobic or anaerobic growth, was used as a normalization control. As shown in Fig. 4, expression of *zipA* gene was ∼3-fold lower in PAO1 grown by anaerobic respiration than in aerobically grown PAO1. A more than 10-fold decrease was observed in transcript levels of both *ftsZ* and *ftsA* during anaerobic vs. aerobic respiration. We then examined transcript levels of *murD* (PA4414) and *murF* (PA4416) genes encoding UDP-N-acetylmuramoylalanine-D-glutamate ligase and UDP-N-acetylmuramoylalanyl-D-glutamyl-2,6-diaminopimelate-D-alanyl-D-alanyl ligase, two important enzymes involved in peptidoglycan synthesis. Likewise, transcript levels of these two genes in PAO1 grown by anaerobic respiration were only ∼13% of those achieved in cells grown by aerobic respiration. This result suggests that expression of genes involved in Z-ring formation and peptidoglycan synthesis, two critical steps for the optimal cell division, is significantly reduced during the anaerobic growth in *P. aeruginosa.*

### Functional nitrite reductase (NIR) is required for anaerobiosis-triggered cell elongation

**Figure 5:**
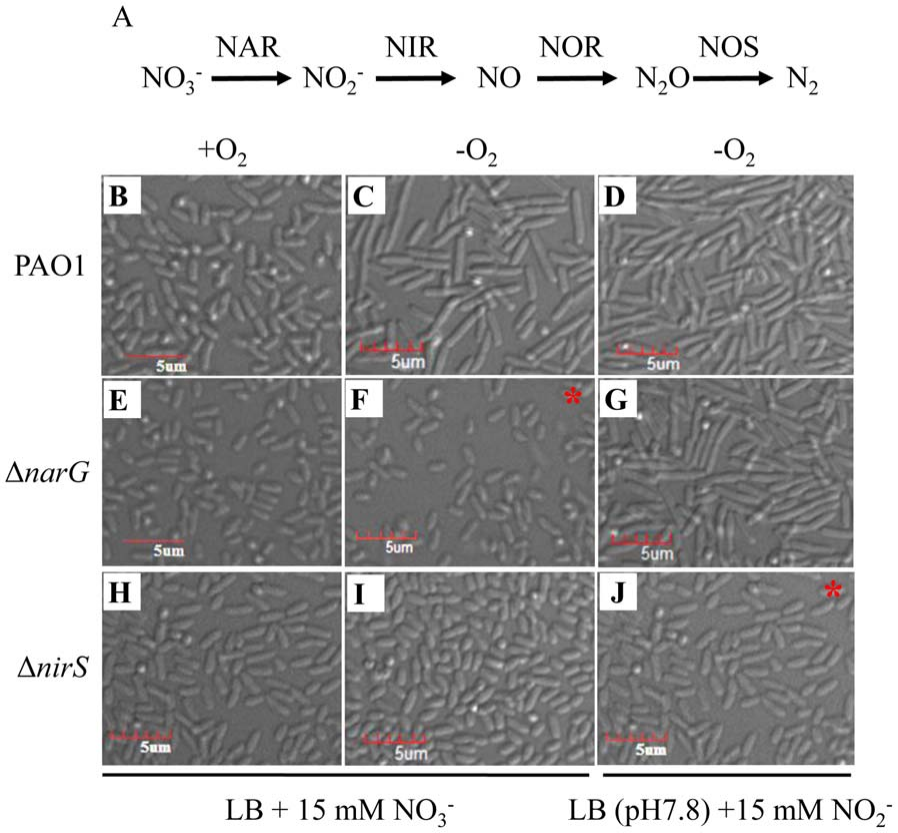
Cell elongation phenotypes of *P. aeruginosa* mutant strains under aerobic and anaerobic growth conditions. (**A**) Anaerobic respiratory (denitrification) pathway. Enzymes involved in each reduction step are termed **n**itr**a**te **r**eductase (NAR), **n**itr**i**te **r**eductase (NIR), **n**itric **o**xide **r**eductase (NOR), and **n**itr**o**u**s** oxide reductase (NOS), respectively. (**B–J**) DIC images of PAO1 (**B**, **C,** and **D**), Δ*narG* (**E**, **F,** and **G**), Δ*nirS* (**H**, **I,** and **J**). Bacterial strains were grown for 18 hours in LB +15 mM NO_3_^−^ aerobically (**B**, **E,** and **H**), anaerobically (**C**, **F,** and **I**), and anaerobically in LB (pH 7.8) +15 mM NO_2_^−^ (**D**, **G**, and **J**). A scale bar of 5 µm is indicated in the bottom left of each panel. * No discernible growth was observed in these two cultures and thus, cells were concentrated for image acquisition.

Anaerobic respiration in *P. aeruginosa* involves a sequential reduction of nitrate (NO_3_^−^) or nitrite (NO_2_^−^) to N_2_ [6]. Higher cell yield was obtained in PAO1 growth using NO_3_^−^ as an electron acceptor than NO_2_^−^-supported anaerobic growth (Fig. S1), suggesting that NO_3_^−^ reduction is the major step that is coupled to ATP synthesis in wild type *P. aeruginosa*. Since cell elongation only occurred in anaerobically respiring *P. aeruginosa*, the anaerobic respiration pathway was further dissected to uncover the crucial step that is responsible for cell elongation. To address this issue, cell elongation phenotypes of Δ*narG* and Δ*nirS* mutant, which became defective in NAR or NIR, respectively, were tested (Fig. 5A). Consistent with findings described in Fig. 1, wild type strain PAO1 and two mutant strains grown by aerobic respiration maintained their regular rod-shape morphology even in the presence of 15 mM NO_3_^−^ (Fig. 5B, E and H). PAO1 anaerobically grown on NO_3_^−^ (Fig. 5C) or NO_2_^−^ (Fig. 5D) was invariably elongated compared to its aerobically grown counterpart (Fig. 5B). This suggests that anaerobic growth supported by either electron acceptor can trigger cell elongation in *P. aeruginosa*. To minimize the growth-inhibitory effect of NO_2_^−^ on bacterial growth [26], the pH of the culture medium was adjusted to 7.8, when NO_2_^−^ is used as an electron acceptor. It was important to note that the Δ*narG* and Δ*nirS* mutants did not grow on NO_3_^−^ or NO_2_^−^, respectively, due to the lack of the enzyme that can reduce the corresponding electron acceptor (Fig. S1) and thus, no cell elongation was observed in each of these two anaerobic cultures (Fig. 5F and J, * denotes no growth). This result further validates that active respiratory growth is the prerequisite for the cell elongation during anaerobic growth. Importantly, Δ*narG* mutant bacteria grown by NO_2_^−^ respiration were as elongated as its parental strain, PAO1 (Fig. 5G), whereas Δ*nirS* mutant cells were not elongated upon anaerobic growth on NO_3_^−^ (Fig. 5I). Final cell density in these two cultures was comparable to each other (Fig. S1), suggesting that the formation of these two contrasting cell shapes (Fig. 5G vs. 3I) was not caused by the differential bacterial cell growth.

Moreover, mucoid *P. aeruginosa*, FRD1, determined to possess undetectable NIR activity [26], was not elongated under the same NO_3_^−^ respiring condition (Fig. S2). Together, these results suggest that NIR-mediated reduction of NO_2_^−^ to NO is the critical step that triggers anaerobiosis-induced cell elongation in *P. aeruginosa*.

### Biofilm formation of the non-elongated Δ*nirS* mutant was significantly reduced under the NO_3_^−^ respiring condition

**Figure 6:**
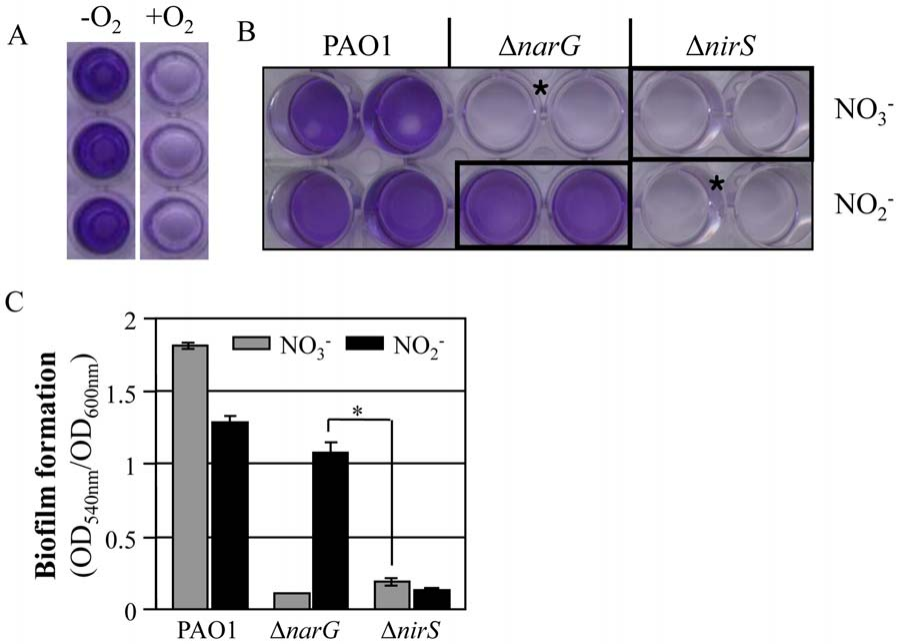
Biofilm formation of the Δ*narG* and Δ*nirS* mutants during anaerobic respiration using NO_3_^−^ or NO_2_^−^ as electron acceptors. (**A**) Crystal violet (CV) staining of biofilms formed by PAO1 under aerobic and anaerobic respiration conditions. (**B**) CV staining of biofilms formed by *P. aeruginosa* strains, PAO1, Δ*narG*, and Δ*nirS*. Strains were grown for 18 hours prior to staining in pH-adjusted LB media containing either 15 mM NO_3_^−^ (top row) or NO_2_^−^ (bottom row). CV staining was performed as described in materials and methods. *No discernible growth was observed in these two cultures. (**C**) Quantification of the CV staining. OD_540 nm_ values were normalized with cell mass measured by OD_600 nm_. *p<0.001 vs. biofilms formed by the Δ*nirS* mutant.

It has been previously shown that during anaerobic respiration, *P. aeruginosa* formed significantly more robust biofilm compared to when bacteria grow aerobically [17], [18]. This finding was successfully reproduced in our crystal violet biofilm staining assay as shown in Fig. 6A. Since elongated cell morphology was only observed under anaerobic respiration condition, we sought to examine if this enhanced biofilm formation is caused by a modified cell biological feature associated with cell elongation. To address this question, the biofilm formation of Δ*narG* and Δ*nirS* mutants that showed distinct cell elongation phenotypes were compared (Fig. 5G and I). pH-buffered L-Broth media was used to maximize bacterial growth by NO_2_^−^ respiration. Wild type PAO1 formed very robust biofilm in both NO_3_^−^ and NO_2_^−^-stimulated anaerobic growth (Fig. 6B). Quantification analysis of biofilm formation by measuring OD_540 nm_/OD_600 nm_, however, indicated that denser biofilm was formed under the condition of NO_3_^−^ respiration than NO_2_^−^ respiration (Fig. 6C). This was likely due to greater bacterial growth by NO_3_^−^ respiration (Fig. S1). It is important to note that the Δ*narG* mutant, which was elongated during anaerobic growth using NO_2_^−^ as an electron acceptor (Fig. 5G), formed biofilm that was almost as robust as the PAO1 biofilm under the same growth conditions (Fig. 6B). In contrast, biofilm formation was completely abrogated in the non-elongated Δ*nirS* mutant upon NO_3_^−^ respiration (Fig. 6B). Again, this distinct biofilm formation was not due to the differential growth of these two anaerobic cultures, because cell growth of the Δ*nirS* mutant on NO_3_^−^ was slightly greater than the Δ*narG* mutant in NO_2_^−^ (Fig. S1). As expected, anaerobic cell growth of Δ*narG* on NO_3_^−^ or Δ*nirS* on NO_2_^−^ was minimal and thus, no biofilm was formed in each of these two cultures. These results suggest that anaerobiosis-induced cell elongation, which is dependent on the presence of NIR activity, plays a critical role in robust biofilm formation under these conditions.

### Cell elongation and biofilm formation were both suppressed in the presence of Carboxy-PTIO, a stoichiometric scavenger in PAO1

**Figure 7:**
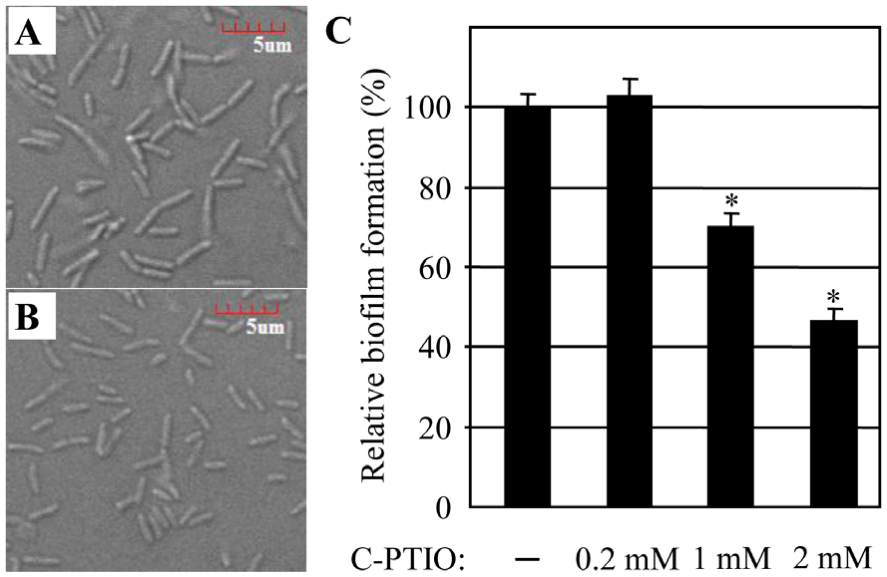
Effects of exogenously amended carboxy-PTIO on cell elongation and anaerobic biofilm formation in PAO1. DIC images of PAO1 grown without (**A**) or with (**B**) 2 mM carboxy-PTIO. Cells were grown in LB containing 0.4% NO_3_^−^ under the anaerobic condition. Images were acquired and processed as described in Fig. 1 and 5. (**C**) The effect of carboxy-PTIO on the anaerobic biofilm formation. PAO1 was grown in the absence or presence of increasing concentrations of carboxy-PTIO for 18 hours and biofilm formed in each culture was stained with CV. *p<0.001 vs. biofilms formed in the presence of 2 mM or 1 mM carboxy-PTIO.

Although nitric oxide (NO), the product of NIR, is further reduced to N_2_O, steady-state level of nitric oxide (NO) is maintained during anaerobiosis [16], [34]. Since (i) NIR activity, which generates NO as its product, is required for cell elongation as shown in Fig. 5, and (ii) the enzymatic action of NIR is the only source of NO production under anaerobic growth conditions [6], it was postulated that production of intracellular NO may account for the anaerobiosis-induced cell elongation. To address this notion, the cell elongation phenotype of PAO1 grown in the presence of 2 mM carboxy-PTIO, a stoichiometric NO scavenger was tested. In our previous work, carboxy-PTIO, which penetrates the periplasm and scavenges NO before it escapes to the cytoplasm or is reduced by NOR, successfully protected *P. aeruginosa* strains from NO-mediated intoxication [17]. As shown in Fig. 7A and B, cell elongation was suppressed in the presence of carboxy-PTIO (4.87±1.89 µm vs. 2.35±0.54 µm).

Results described in Fig. 6 indicate that cell elongation is an important cellular event that contributes to the biofilm formation under the condition of anaerobic respiration. To further prove the effect of cell elongation on biofilm formation, biofilm formation in a condition where cell elongation was hindered by the addition of carboxy-PTIO was examined. As shown in Fig. 7C, a dose-dependent decrease in biofilm formation was detected in the presence of increasing amount of carboxy-PTIO. Upon growth with 2 mM carboxy-PTIO, biofilm robustness was ∼47% of that of the control anaerobic biofilm suggesting that the perturbation of cell elongation exerted an adverse effect on biofilm formation. No adverse effect of added carboxy-PTIO on cell viability was observed up to 2 mM concentration (data not shown).

### Clump formation was induced during anaerobic growth

**Figure 8:**
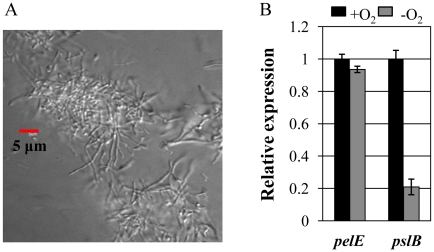
Bacterial clump formation during anaerobic growth and qRT-PCR analysis of genes involved in polysaccharide biosynthesis. (**A**) A DIC image of clumps observed in a planktonic anaerobic culture of PAO1. A scale bar of 5 µm is shown. (**B**) qRT-PCR was conducted on cDNA synthesized from 2 µg total RNA extracted from PAO1 grown either aerobically (black bars) or anaerobically (gray bars). Assay conditions were identical as described in Fig. 4. Three independent experiments were performed and values of mean ± SEM are displayed in each bar.

Importantly, we often observed highly cohesive cell clusters in the elongated *P. aeruginosa*. Fig. 8A shows a representative image of clusters formed by PAO1 during an anaerobic planktonic culture. When we attempted to quantify the cluster formation in anaerobic vs. aerobic cultures of PAO1, ∼30 (±14) clusters per 20 µl of overnight culture that was mounted on the slide glass were identified in our microscopic analysis. Similar clusters were detected neither in the aerobic *P. aeruginosa* cultures nor anaerobic Δ*nirS* mutant culture (data not shown). This data strongly suggest that cell elongation that specifically occurs during anaerobic respiration is involved in the cohesive clump formation that eventually leads to the robust biofilm formation.

Two distinct genetic loci, *pel* and *psl* produce carbohydrate-rich biofilm matrix that can hold bacterial cells together [35], [36], [37]. Therefore, it is of interest to examine whether Pel and/or Psl polysaccharide matrix could contribute to the anaerobiosis-induced clump formation. As a way to address this issue, we performed qRT-PCR on *pelE* and *pslB*, genes representing each of these two clusters. Shown in Fig. 8B is the transcriptional modulation of these two genes under aerobic vs. anaerobic growth conditions. Transcription of *pelE* gene was not changed upon anaerobic growth, while the transcript level of *pslB* gene was decreased to ∼20% of what was observed in cells grown aerobically. This result suggested that levels of Pel and/or Psl polysaccharides produced during anaerobic respiration would not be higher than those produced during aerobic growth and thus, anaerobiosis-induced enhancement of clump formation is not likely to be associated with altered production of extracellular polysaccharides.

### Biofilm formation was promoted in the presence of carbenicillin that induced cell filamentation

**Figure 9:**
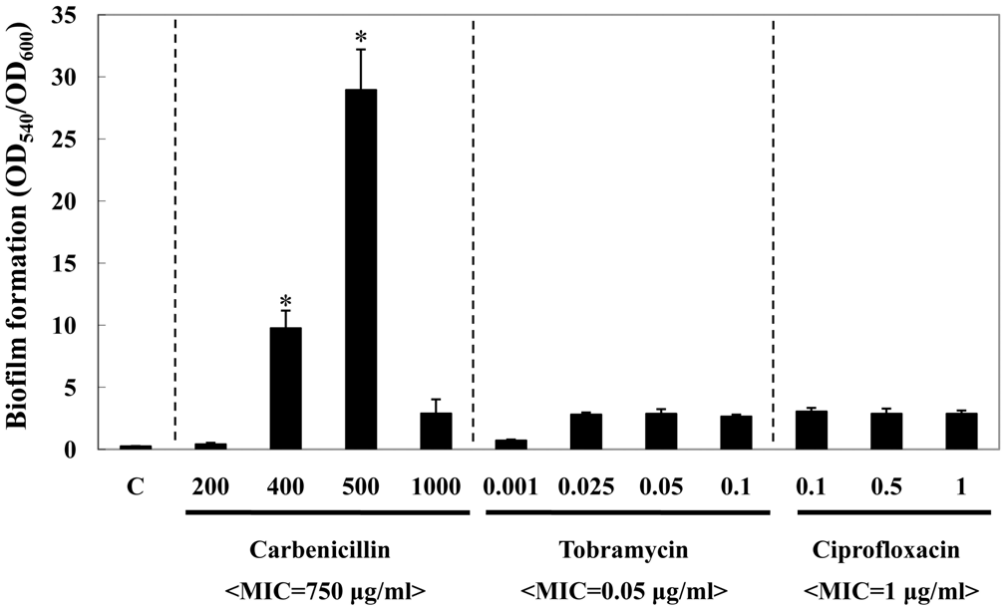
Effects of carbenicillin-induced cell elongation on the biofilm formation in PAO1. Quantification of CV staining of PAO1 biofilms grown with increasing concentrations of carbenicillin, tobramycin or ciprofloxacin (indicated at the bottom). PAO1 was inoculated in antibiotic-containing LB media placed in 96-well plates and grown for 18 hrs aerobically. C: LB only control. *p<0.001 vs. biofilms formed by PAO1 in the presence of tobramycin or ciprofloxacin.

Our results suggest that robust anaerobic biofilm formation is likely mediated by modified cellular features associated with anaerobiosis-specific cell elongation in *P. aeruginosa*. To provide further evidence that supports the correlation between cell elongation and biofilm formation in *P. aeruginosa*, we tested if biofilm formation can also be promoted by other stimulus that causes cell elongation. Because cell elongation occurs upon treatment with carbenicillin in *P. aeruginosa* [38], [39], [40], we compared biofilm formation of PAO1 treated with carbenicillin versus ciprofloxacin and tobramycin. As shown in Fig. 9, biofilm formation of PAO1 was significantly increased in the presence of sublethal doses of carbenicillin. In our microscopic analysis, a high degree of cell filamentation was also observed confirming results from aforementioned referenced studies (data not shown). It is of note that PAO1 was highly resistant to carbenicillin and MIC was determined to be 750 µg/ml, a value consistent with previous findings [40], [41]. In contrast, only a mild increase in biofilm formation was observed in PAO1 treated with tobramycin or ciprofloxacin (Fig. 9). No cell shape change was observed in aerobic growth with each of these two antibiotics (data not shown). This result demonstrated that treatment with an antibiotic that specifically induced cell elongation also promoted biofilm formation further suggesting the positive influence of cell elongation on biofilm formation in *P. aeruginosa*.

## Discussion

Biofilm is a microbial community grown as an aggregate or on a surface with distinct architecture [23]. Biofilm research using *P. aeruginosa* as a model organism has been performed using *in vitro* biofilm grown under aerobic respiration. Although the presence of local regions with reduced oxygen potential has been proposed to exist inside bacterial biofilm [42], [43], previous works showed that anaerobically growing *P. aeruginosa* formed significantly robust biofilm compared with bacteria growing by aerobic respiration [17], [18]. This observation is of clinical importance because the airway of chronic CF patients was suggested to be anaerobic due to the accumulation of abnormally thickened and viscous mucus on top of the airway epithelium [8]. In addition, anaerobically growing *P. aeruginosa* exhibited higher resistance to a range of currently used antibiotics than their aerobically grown counterparts [11], further suggesting that *P. aeruginosa* proliferates inside the patient airway by employing two different modes of antibiotic-resistant growth, i.e. biofilm and anaerobiosis.

The molecular mechanisms behind the enhanced biofilm formation under anaerobic growth conditions are not clearly defined. Here, a unique morphological feature of *P. aeruginosa* grown by anaerobic respiration was identified for the first time and its effect on biofilm formation was investigated. Our results revealed that anaerobic growth of *P. aeruginosa* is concurrently accompanied by abnormally altered cell division. PAO1 grown during anaerobic respiration was ∼5-fold more elongated than aerobically grown cells by a mechanism associated with defective cell division. In *P. aeruginosa*, cell elongation was reported to be caused by nutrient deprivation [44]. It was postulated that bacteria elongate to increase their nutrient uptake as a part of their adaptation process for starvation. However, it seems unlikely that anaerobiosis-induced cell elongation occurred for a similar reason since (i) the cells were grown in rich media (i.e. L-Broth) and (ii) the density at which the culture was harvested for the microscopic image was as low as OD_600_ of ∼0.5. In addition, NO_3_^−^-supported anaerobic growth is considerably luxuriant and thus the final density of an anaerobic culture of wild type PAO1 supplemented with 100 mM NO_3_^−^ was almost comparable with that of aerobic culture (data not shown).

Cell elongation (or filamentation) was also reported to take place under nonpermissive conditions, such as high growth temperature [45], the treatment with certain antibiotics [46] and the UV irradiation [32]. But, bacterial growth under these highly stressful conditions was completely ceased and furthermore, the level of cell elongation appeared to be significantly greater than what was observed in the present study. This suggests that although *P. aeruginosa* favors the utilization of oxygen to generate energy and can grow faster aerobically, cells are not placed under any stress condition in the absence of oxygen and thus, the anaerobiosis-induced cell elongation occurs within a range that is permissive for cell growth.

Our subsequent experiments uncovered the fact that cell elongation was mediated by NO, produced consistently as a spontaneous intermediate of anaerobic respiration [16], [17]. Our previous work [6] and a work by Cork and Poole using *E. coli* as a test organism [47] indicated that NIR activity was necessary to produce NO under anaerobic conditions. Consistent with this finding, the Δ*nirS* mutant that was devoid of NIR activity was not elongated under NO_3_^−^ respiration conditions (Fig. 5). These findings were further proven by the observation that NO-triggered cell elongation was suppressed by the addition of membrane-penetrable carboxy-PTIO (Fig. 7).

During airway infection, *P. aeruginosa* encounters significant level of neutrophils infiltrated into the airway mucus. Along with macrophages and airway epithelial cells, neutrophils produce NO as an important defense molecule [48]. Because our results suggest that elongated cells form more robust biofilm, an interesting hypothesis would be whether *P. aeruginosa* responds to host-derived NO during the infectious process in order to change its cell shape. To test this idea, we have attempted to see the effect of exogenously added NO via acidified NO_2_^−^ [26] on the cell shape change in PAO1 under aerobic growth condition, but no morphological change in response to NO was observed (data not shown). This may suggest that NO-triggered cell elongation only occurs under anaerobic respiration condition using endogenously produced metabolic NO. More experiments are necessary to precisely determine the effect of NO on the bacterial cell shape change.

Our results also demonstrated that the elongated cells are inclined to form highly cohesive clumps, which we believe accounts for the robust biofilm formation under anaerobic condition (Fig. 8). Because clump formation was not observed in non-elongated mutant strains (Δ*nirS* mutant) and aerobically grown *P. aeruginosa*, this finding suggests that anaerobiosis-induced cell elongation likely caused changes in membrane properties that result in the clump formation. Potential questions to be addressed in the future include; (i) how different the cell wall composition of elongated cells is from rod-shaped *P. aeruginosa* and (ii) how the endogenously produced NO can signal a mechanism by which cells undergo elongation.

The discovery of anaerobiosis-induced morphological change inspired the present study to test its effect on biofilm formation. Since (i) cell-to-cell contact, which is an important determinant for biofilm formation, can be facilitated by an enlarged cell surface and (ii) highly cohesive autoaggregates were only detected in elongated cells, it was hypothesized that the robust anaerobic biofilm formation might result from the elongated cell shape. To address this important question, the elongation-defective mutant(s) were subjected to isolation. However, a genome-wide mutant library screen to isolate such mutants was not feasible, due to the lack of an efficient assay system that would allow us to isolate the defective mutants. Alternatively, the effect of the disruption of genes involved in the anaerobic respiration pathway on the elongation phenotype was examined since cell elongation occurred as a consequence of anaerobic respiration in wild type PAO1 (Fig. 1). The results described in Fig. 5 demonstrated that the Δ*nirS* mutant remained rod-shaped, while the Δ*narG* mutant was elongated as PAO1 under anaerobic growth. This finding provided us with an opportunity to compare the biofilm forming capability of the mutants that were in marked contrast to each other in their cell morphology.

NIR was also reported to be required for the type III secretion system and virulence towards human monocyte cell line THP-1 and *Caenorhabditis elegans* even in conditions where oxygen is present [7], [49]. This suggests that NIR is likely important in *P. aeruginosa* pathogenesis irrespective of the environmental oxygen concentration. Results presented in the current study, however, clearly demonstrated the role of NIR in anaerobiosis-specific condition. Our conclusion that enhanced biofilm formation occurred under anaerobic condition was due, at least in part, to the NIR-mediated cell elongation and was based on the following results; (i) the non-elongated Δ*nirS* mutant did not form biofilm under NO_3_^−^ respiration conditions (Fig. 6), (ii) the Δ*narG* mutant grown by NO_2_^−^ respiration was highly elongated and formed very robust biofilm (Fig. 6), and (iii) biofilm formation was decreased by the addition of carboxy-PTIO that inhibited cell elongation (Fig. 7).

In summary, this report provides initial insights into the cell shape-dependent biofilm-forming characteristics of *P. aeruginosa*. Anaerobic biofilm formation that represents a persistent bacterial survival mechanism inside the patient airway requires the activity of NIR. Therefore, it is suggested that this key player could serve as a target, inhibition of which can be beneficial as novel anti-*pseudomonas* therapeutic interventions are further developed.

## Supporting Information

Figure S1 Final cell density of anaerobic cultures of three *P. aeruginosa* strains. Bacterial strains were grown for 18 hours in LB +15 mM NO_3_^−^ (black bars) or NO_2_^−^ (gray bars) anaerobically. For a negative control, PAO1 was grown in plan LB. * p<0.01 vs. growth with NO_3_^−^.
(TIF)

Figure S2 DIC images of anaerobically grown PAO1 and FRD1. A scale bar of 5 µm is indicated in the top of each panel.
(TIF)

Table S1 Primers used for qRT-PCR.
(DOCX)
