# Supplementary figures and images for: Contribution of Cell Elongation to the Biofilm Formation of Pseudomonas aeruginosa during Anaerobic Respiration
Source: PLoS One. 2011 Jan 18;6(1):e16105. doi: 10.1371/journal.pone.0016105 (PMC3022656; doi:10.1371/journal.pone.0016105)

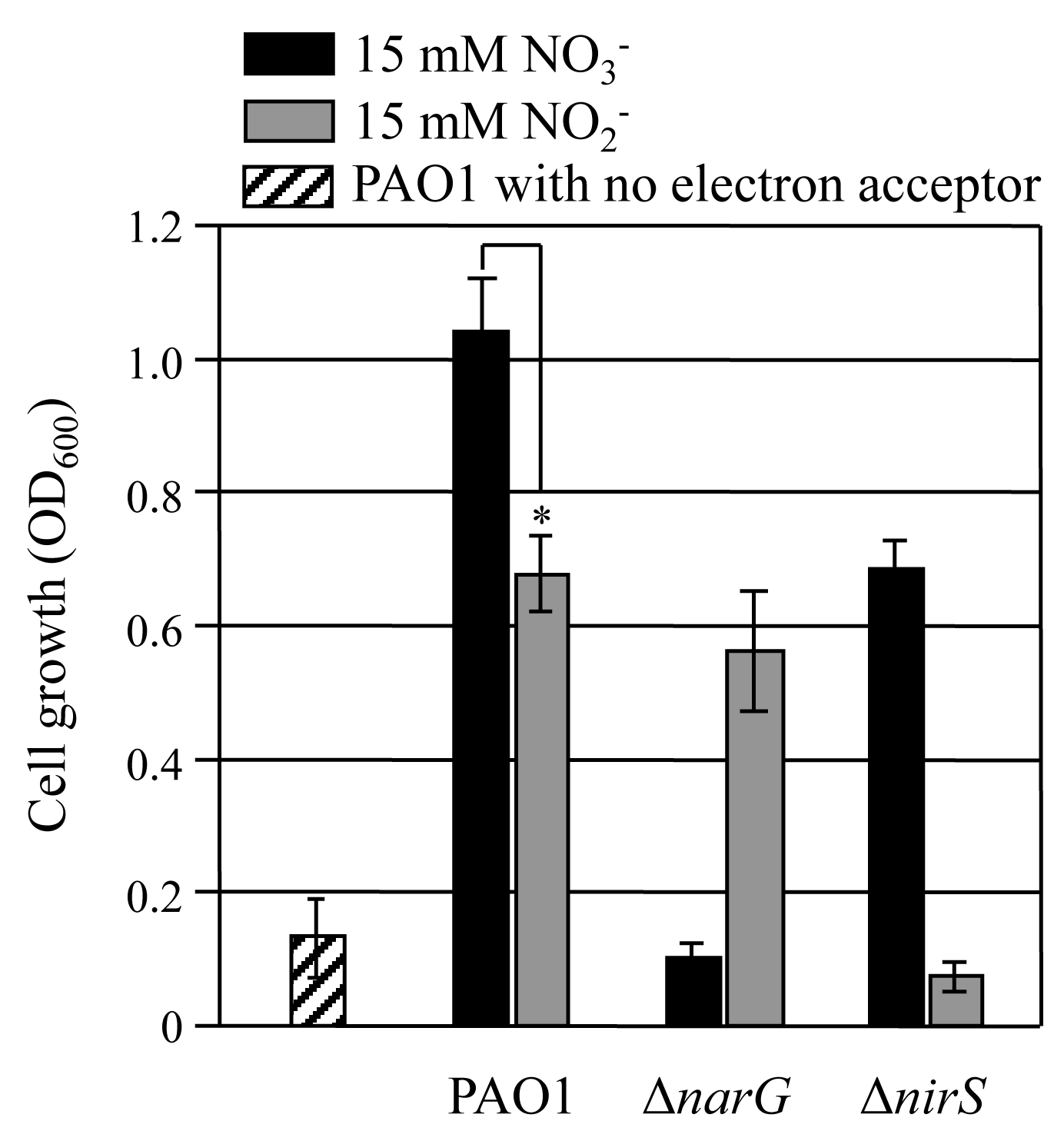

Supplement: Figure S1 — Final cell density of anaerobic cultures of three P. aeruginosa strains. Bacterial strains were grown for 18 hours in LB +15 mM NO3 − (black bars) or NO2 − (gray bars) anaerobically. For a negative control, PAO1 was grown in plan LB. * p<0.01 vs. growth with NO3 −. (TIF) [file pone.0016105.s001.tif]

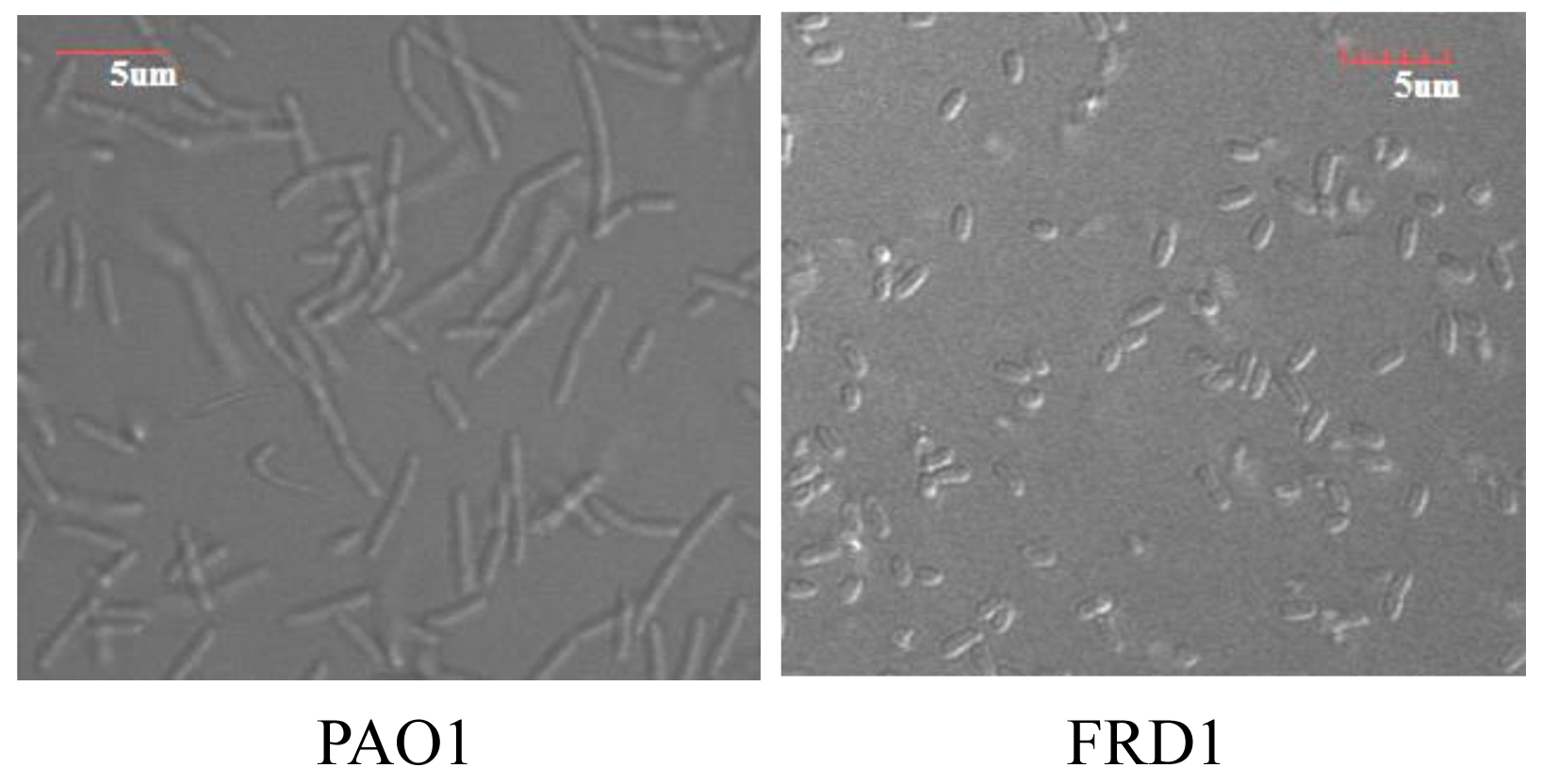

Supplement: Figure S2 — DIC images of anaerobically grown PAO1 and FRD1. A scale bar of 5 µm is indicated in the top of each panel. (TIF) [file pone.0016105.s002.tif]
