# Supplementary material for: Contribution of Cell Elongation to the Biofilm Formation of Pseudomonas aeruginosa during Anaerobic Respiration
Source: PLoS One. 2011 Jan 18;6(1):e16105. doi: 10.1371/journal.pone.0016105 (PMC3022656; doi:10.1371/journal.pone.0016105)
Supplement: Table S1 — Primers used for qRT-PCR. (DOCX) [file pone.0016105.s003.docx]

| Gene name | Primer sequences (5'-3') |
| --- | --- |
| *zipA* | F : AAGAGCGGGAGAAAGCCCCT  R : CGGGCGATGACGTTGATGAT |
| *ftsZ* | F : GCCGAAGTGGCGAAGGAAAT  R : GGTGATCAGCGAATCGACGC |
| *ftsA* | F : CGACGAGCTGTTCACCCTGG  R : GCGCCTTCCATCTTCGAGGT |
| *murD* | F : CGAACTGAAGATCCGTGGCG  R : GATGAGCCAGGCCGGAAAAC |
| *murF* | F : AAGATCGTCGAGGCGAAGGG  R : CGTCCAGGTTCAGTACGGCG |
| *pslB* | F : TGATCATCGTCGACACCCCC  R : TTCCTGGGCGACGAACTTGA |
| *pelE* | F : ATCCCGATCCTCAAGCTGGC  R : CTCGATGCGCTGGTTGATCC |
| *rpoD* | F : AAGGCCCTGAAGAAGCACGG  R : GATCGGCATGAACAGCTCGG |

Table S1. Primers used for qRT-PCR.
